# Supplementary material for: Metapopulation ecology links antibiotic resistance, consumption, and patient transfers in a network of hospital wards
Source: eLife. 2020 Oct 27;9:e54795. doi: 10.7554/eLife.54795 (PMC7690951; doi:10.7554/eLife.54795)
Supplement: Supplementary file 1. — (a) Summary statistics (mean, interquartile range) of connectivity for each variant. (b) Comparison of log-likelihood values from multivariable Poisson and negative binomial regressions and overdispersion parameters from quasi-Poisson multivariable regression of the incidence of infections with 17 pathogen variants. All models included total antibiotic consumption, connectivity, ward size, ward type, and the incidence control value as variables. [file elife-54795-supp1.docx]

Metapopulation ecology links antibiotic resistance, consumption and patient transfers in a network of hospital wards

Julie Teresa Shapiro^1^, Gilles Leboucher^2^, Anne-Florence Myard-Dury^3^, Pascale Girardo^4^, Anatole Luzatti^4^, Mélissa Mary^4^, Jean-François Sauzon^4^, Bénédicte Lafay^5^, Olivier Dauwalder^4^, Gérard Lina^1,4^, Christian Chidiac^6^, Sandrine Couray-Targe^3^, François Vandenesch^1,4^, Jean-Pierre Flandrois^5^, Jean-Philippe Rasigade^1,4*^

^1^Centre International de Recherche en Infectiologie, CIRI INSERM U1111, University of Lyon, France.

^2^Département de Pharmacie, Hospices Civils de Lyon, Lyon, France.

^3^Pôle de Santé Publique, Département d'Information Médicale, Hospices Civils de Lyon, Lyon, France.

^4^Institut des Agents Infectieux, Hospices Civils de Lyon, Lyon, France.

^5^Laboratoire de Biométrie et Biologie Evolutive, UMR CNRS 5558, University of Lyon, France.

^6^Service des Maladies Infectieuses et Tropicales, Hospices Civils de Lyon, Lyon, France.

**Supplementary File 1**

**Table 1a. Summary statistics (mean, interquartile range) of connectivity for each variant.**

| **Taxon** | **Resistance profile** | **Acronym** | **Mean Connectivity (Interquartile Range)** |
| --- | --- | --- | --- |
| *Escherichia coli* | Susceptible to 3GC and carbapenems | EC | 168.2 (16.2, 177.3) |
|  | 3GC-resistant | 3GCREC | 11.8 (1.0, 12.2) |
|  | Carbapenem-resistant | CREC | 0.15 (0.003, 0.13) |
| *Klebsiella pneumoniae* | Susceptible to 3GC and carbapenems | KP | 20.6 (1.8, 21.3) |
|  | 3GC-resistant | 3GCRKP | 5.5 (0.4, 6.0) |
|  | Carbapenem-resistant | CRKP | 0.21 (0.0, 0.23) |
| *Enterobacter cloacae* complex | Susceptible to 3GC and carbapenems | EB | 4.9 (0.3, 4.3) |
|  | 3GC-resistant | 3GCREB | 1.5 (0.1, 1.6) |
|  | Carbapenem-resistant | CREB | 0.74 (0.03, 0.73) |
| *Pseudomonas aeruginosa* | Carbapenem-susceptible | PA | 19.8 (1.7, 20.3) |
|  | Carbapenem-resistant | CRPA | 3.9 (0.2, 4.4) |
| *Acinetobacter baumannii* | Carbapenem-susceptible | AB | 1.7 (0.1, 1.3) |
|  | Carbapenem-resistant | CRAB | 0.69 (0.0, 0.3) |
| *Enterococcus faecium* | Vancomycin-susceptible | EF | 3.5 (0.1, 3.6) |
|  | Vancomycin-resistant | VREF | 0.09 (0.00, 0.02) |
| *Staphylococcus aureus* | Methicillin-susceptible | SA | 273 (5.9, 67.2) |
|  | Methicillin-resistant | MRSA | 151 (0.5, 6.4) |

**Table 1b. Comparison of log-likelihood values from multivariable Poisson and negative binomial regressions and overdispersion parameters from quasi-Poisson multivariable regression of the incidence of infections with 17 pathogen variants. All models included total antibiotic consumption, connectivity, ward size, ward type, and the incidence control value as variables.**

| Variant | Poisson loglik | Negative binomial loglik | Quasi-Poisson Theta |
| --- | --- | --- | --- |
| EC | -917.4 | -908.1 | 1.26 |
| 3GCREC | -509.4 | -502.2 | 1.21 |
| CREC | -65.5 | -65.5 | 0.87 |
| KP | -550.6 | -550.6 | 0.99 |
| C3GRKP | -432.7 | -427.2 | 1.31 |
| CRKP | -96.6 | -96.4 | 0.68 |
| EB | -323.2 | -322.3 | 1.67 |
| 3GCREB | -274.5 | -274.1 | 1.13 |
| CREB | -190.2 | -190.1 | 1.03 |
| PA | -555.2 | -552.8 | 1.13 |
| CRPA | -371.1 | -359.5 | 1.80 |
| AB | -179.2 | -175.9 | 1.20 |
| CRAB | -28.6 | -28.6 | 0.25 |
| EF | -433.2 | -388.9 | 2.51 |
| VREF | -23.6 | -23.6 | 0.74 |
| SA | -761.1 | -706.1 | 1.67 |
| MRSA | -387.6 | -380.6 | 1.45 |
